# Efficacy and tolerability of peg-only laxative on faecal impaction and chronic constipation in children. A controlled double blind randomized study vs a standard peg-electrolyte laxative

**DOI:** 10.1186/1471-2431-12-178

**Published:** 2012-11-15

**Authors:** Francesco Savino, Serena Viola, Maiullari Erasmo, Giovanni Di Nardo, Salvatore Oliva, Salvatore Cucchiara

**Affiliations:** 1 Department of Pediatrics 1, “Regina Margherita” Children’s Hospital, University of Turin, Azienda Ospedaliera Città della Salute e della Scienza della Città di Torino, Turin, Italy; 2 Department of Suergery Pediatrics, “Regina Margherita” Children’s Hospital, Azienda Ospedaliera Città della Salute e della Scienza della Città di Torino, Turin, Italy; 3 Pediatric Gastroenterology and Liver Unit, “La Sapienza” University of Rome, Rome, Italy

**Keywords:** Constipation, Laxatives, Children, Polyethylene glycol, Macrogol

## Abstract

**Background:**

PEG-based laxatives are considered today the gold standard for the treatment of constipation in children. PEG formulations differ in terms of composition of inactive ingredients which may have an impact on acceptance, compliance and adherence to treatment. We therefore compared the efficacy, tolerability, acceptance and compliance of a new PEG-only formulation compared to a reference PEG-electrolyte (PEG-EL) formulation in resolving faecal impaction and in the treatment of chronic constipation.

**Methods:**

Children aged 2–16 years with functional chronic constipation for at least 2 months were randomized to receive PEG-only 0.7 g/kg/day in 2 divided doses or 6.9 g PEG-EL 1–4 sachets according to age for 4 weeks. Children with faecal impaction were randomized to receive PEG-only 1.5/g/kg in 2 divided doses until resolution or for 6 days or PEG-EL with an initial dose of 4 sachets and increasing 2 sachets a day until resolution or for 7 days.

**Results:**

Ninety-six children were randomized into the study. Five patients withdrew consent before starting treatment. Three children discontinued treatment for refusal due to bad taste of the product (1 PEG-only, 2 PEG-EL); 1 (PEG-EL) for an adverse effect (abdominal pain). Intent-to-treat analysis was carried out in 49 children in the PEG-only group and 42 in the PEG-EL group.

No significant differences were observed between the two treatment groups at baseline.

Adequate relief of constipation in terms of normalized frequency and painless defecation of soft stools was achieved in all patients in both groups. The number of stools/week was 9.2 ± 3.2 (mean ± SD) in the PEG-only group and 7.8 ± 2.4 in the PEG-EL group (p = 0.025); the number of days with stool was 22.4 ± 5.1 in the PEG-only group and 19.6 ± 7.2 in the PEG-EL group (p = 0.034).

In the PEG-only group faecaloma resolution was observed in 5 children on the second day and in 2 children on the third day, while in the PEG-EL group it was observed in 2 children on the second day, in 3 children on the third day and in 1 child on the fifth day.

Only 2 patients reported mild treatment-related adverse events: 1 child in the PEG-only group had diarrhoea and vomiting and 1 child in the PEG-EL group had abdominal pain requiring treatment discontinuation. The PEG-only preparation was better tolerated as shown by the lower frequency of nausea than in the PEG-EL group.

In the PEG-only group, 96% of patients did not demonstrate any difficulties associated with treatment, as compared with 52% of patients in the PEG-EL group (p < 0.001). Also, the PEG-only formulation taste was better than that of PEG-EL (p < 0.001). The difference between the percentage of subjects who took > 80% of the prescribed dose was in favour of the PEG-only group (98% *vs*. 88%), though it did not reach a conventional statistical level (p = 0.062).

**Conclusion:**

PEG-only was better tolerated and accepted than PEG-EL in children with chronic constipation. At the higher PEG doses recommended by the manufactures children in the PEG-only group had higher and more regular soft stool frequency than PEG-EL.

**Trial registration:**

ClinicalTrials.gov: NCT01592734

## Background

Constipation is a very common childhood complaint. The condition is chronic in more than one third of patients and is a common reason for referral to secondary care [1,2].

In 90-95% of children, constipation is functional, which means that there is no objective evidence of a pathological condition [3].

Some factors leading to constipation such as inadequate daily fiber intake, insufficient fluid intake, and poor physical activity are considered to be modifiable. The most frequent cause in children is development of a withholding behaviour after experiencing a painful or frightening evacuation [4].

Painful defecation, in fact, is considered a common trigger to faecal retention leading to a cycle of fear and further retention. Retention of faeces can lead to prolonged faecal stasis in the colon, with reabsorption of fluids and increase in the size and consistency of stools. It is often necessary to use a laxative therapy to achieve comfortable defecation in constipated children.

Polyethylene glycol solution (PEG, or macrogol according to the international non-proprietary name) is an osmotic laxative agent that is absorbed in only trace amounts from the gastrointestinal tract. Nowadays, it is routinely used to treat chronic constipation in adults [5,6].

PEG-based laxatives have been shown to be effective and safe for chronic constipation and for resolving faecal impaction in children [7-12].

Different PEG-based laxatives with [13-17] or without [18-20] electrolytes are available on the market. All PEG-based formulations have been shown in placebo-controlled and active comparator trials to be safe and effective in the treatment of chronic constipation in children. However, there is insufficient information regarding the comparison of other features such as tolerability, palatability and ease of administration [21] between PEG formulations which may influence adherence and, in turn, provide better constipation management in children.

A new PEG 4000-only laxative with no excipients or flavourings in a tasteless, odourless powder that can be mixed with water or any other common beverage has been recently introduced in the market.

The aim of this randomized study was to compare the efficacy, tolerability, acceptance and compliance of a new PEG-only formulation compared to a reference PEG-electrolyte formulation in resolving faecal impaction and in the treatment of chronic constipation.

## Methods

### Study design

This was a randomized, single-blind, parallel group study of a PEG-only laxative vs. a PEG-electrolyte laxative in the resolution of faecal impaction and the treatment of chronic constipation.

The study was reviewed by the hospital ethical committee and was carried out in accordance to Good Clinical Practice and Declaration of Helsinki. The investigators obtained a signed informed consent before patient enrolment.

### Patients

Children were eligible if they were aged between 2 and 16 years and had a diagnosis of functional constipation according to Rome III criteria or showed faecal impaction at physical examination. Symptoms of functional constipation should have been present for at least 2 months.

Exclusion criteria included children with organic causes of defecation disorders, such as Hirschsprung disease, spina bifida, hypothyroidism, or other metabolic or renal abnormalities; children receiving medication influencing gastrointestinal motility; children with suspected gastrointestinal obstruction or stenosis.

### Study products

PEG-only laxative (Onligol® powder for oral solution, 400-g bottle with measuring spoon, Promefarm srl, Italy).

Faecal impaction: 1.5 g/kg/day divided in 2 doses until resolution or for max 6 days (fixed dose).

Constipation: in children <20 kg 0.7 g/kg/day; in children >20 kg same daily dose with a maximum limit of 30 g daily. The daily dose was divided in 2 administrations. The duration of treatment was 4 weeks.

PEG-electrolyte (Movicol® Bambini powder for oral solution, 6.9 g sachets (Norgine, Italy).

Faecal impaction: increasing dose up to resolution or up to a 7-day treatment plan as follows (day 1: 4 sachets; day 2: 6 sachets; day 3: 8 sachets; day 4: 10 sachets; day 5: 12 sachets; day 6: 12 sachets; day 7: 12 sachets).

Constipation: 1 sachet in children aged 2–6 years; 2 sachets in children aged 7–11 years; 4 sachets in children aged 12–16 years.

### Randomization and blinding

To ensure a balanced allocation of treatments among different ages, separate computer generated randomized lists were used for the 3 age groups (2–5 years, 6–11 years, 12–16 years). Because of obvious differences in the treatment appearance and taste, blinding of the participants and part of study personnel was not possible. The doctor who performed the evaluation was not involved in the allocation of treatment and remained blinded as to the type of treatment received by patients during the study.

### Study program

– **Visit 1** (Enrolment, -7/-10 days)

At enrolment, before receiving any intervention, caregivers of patients provided information on demographics and medical history, and a physical examination including rectal digital examination (if needed) was performed. A bowel diary was delivered to the patient and he/she had to stop laxative intake. In case of faecal impaction treatment was initiated.

– **Visit 2** (T0)

During the second visit, the doctor performed an evaluation of bowel diaries and the response to faecal impaction treatment. The patient started the constipation treatment.

– **Visit 3** (+28 days)

During the last visit, bowel diary evaluation was performed. The doctor also assessed tolerability, acceptance and patient compliance.

### Evaluation of efficacy

– **Primary variable:**

Stool frequency over the 4 weeks of treatment.

– **Secondary variables:**

Occurrence and timing of faecal disimpaction, stool consistency, frequency of pain/difficulty in passing stools over 4 weeks, frequency of soiling episodes over 4 weeks, parent and child satisfaction, use of laxative, tolerability (episodes of nausea and abdominal pain), acceptability (palatability 5-point scale and ease of administration), compliance with preparation (> 80% of the prescribed dosage).

### Adverse events

All gastrointestinal and non-gastrointestinal unexpected adverse events which occurred during the study were collected.

### Statistical analysis

Baseline and end-of-treatment characteristics have been summarized using the usual descriptive statistics: by count and percentage for qualitative variables and by mean and standard deviation for quantitative variables.

As regards the efficacy analysis, treatments were compared for each measure by calculating the difference between the values or the medium frequencies observed in the period of treatment.

Treatment comparison was carried out using t-test and chi-square test for quantitative and qualitative variables, respectively. All tests were considered two-tailed with significance level set to 5%; 95% confidence intervals were also measured.

## Results

**Figure 1:**
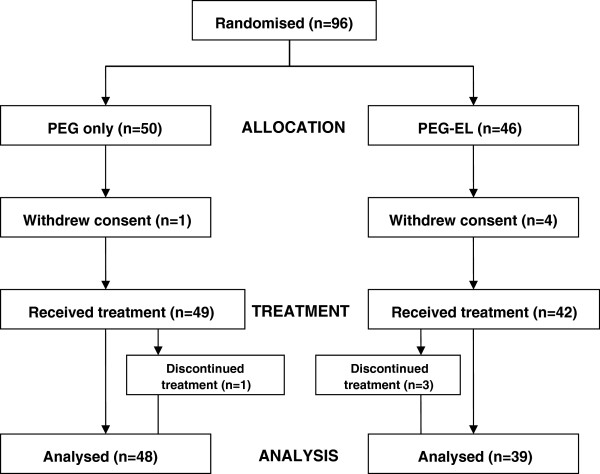
Trial design.

Of the 96 children enrolled, 5 withdrew consent before starting treatment, leaving a total of 91 (Figure 1).

Four children dropped out; 3 of them (1 PEG-only, 2 PEG-EL) due to the bad taste of the product; 1 (PEG-EL) for an adverse effect. Therefore 49 children in the PEG-only group and 42 in the PEG-EL group were included in the intention-to-treat population.

There were no relevant clinical or demographic differences between the two treatment groups at baseline (Table 1).

**Table 1:** Patients demographics

	**PEG-only**	**PEG-EL**
Patients N.	49	42
Age (range)	2-14	2-13
2-5 yrs	30	26
6-11 yrs	16	14
12-16 yrs	3	2
mean ± SD	5.5 ± 3.0	5.6 ± 3.3
Gender		
Male	22	24
Female	27	18
Height (cm)		
Mean ± SD	107 ± 20	107 ± 23
Weight (kg)		
Mean ± SD	20.7 ± 8.8	21.6 ± 11.2

### Efficacy

Adequate relief of constipation in terms of normalized frequency (≥3 per week) and painless defecation of soft stools was achieved in all patients in both groups.

The mean ± SD number of stools/week in the PEG-only and PEG-EL groups, respectively, were 9.2 ± 3.2 *vs*. 7.8 ± 2.4 stools (p = 0.025) while the mean ± SD number of days with stool in the PEG-only and PEG-EL groups, respectively, were 22.4 ± 5.1 *vs*. 19.6 ± 7.2 days (p = 0.034).

Also for the other secondary parameters (painful stools, frequency of abdominal pain, frequency of episodes of soiling, use of stimulant laxative) an advantage for the PEG-only group was observed, although the comparisons did not prove statistically significant (Table 2).

**Table 2:** Efficacy variables

	**PEG-only**	**PEG-EL**
Evaluable patients	49	42
Number of stools per week		
mean ± SD	9.2 ± 3.2	7.8 ± 2.4
	*p*=0.025	
Painful stools		
No. of days	2.3 ± 3.4	3.2 ± 4.0
	*p*=0.240	
Abdominal pain		
No. of days	2.8 ± 3.8	3.9 ± 3.7
	*p*=0.154	
Soiling		
No. of days	0.5 ± 1.2	0.6 ± 0.9
	*p*=0.721	
Use of stimulant laxative (%)	2 (4%)	3 (7%)
	*p*=0.523	
Number of days with stool		
mean ± SD	22.4 ± 5.1	19.6 ± 7.2
	*p*=0.034	

**Figure 2:**
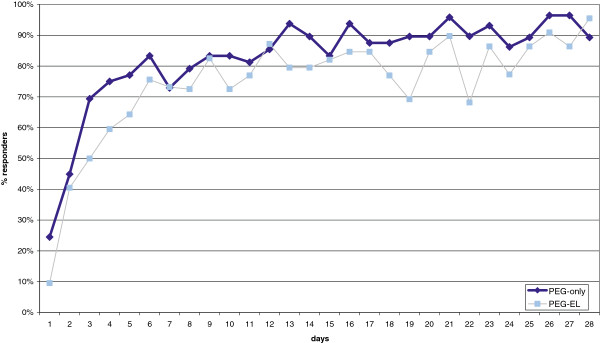
Response to treatment during the study period.

Figure 2 shows the evolution of the response to therapy in both groups during the four weeks (28 days) of treatment.

### Faecal impaction

Faecal impaction was diagnosed in 7 children in the PEG-only group *vs*. 7 children in the PEG-EL group.

In the PEG-only group, faecaloma resolution was observed in 5 children on the second day and in 2 children on the third day, while in the PEG-EL group resolution was achieved in 2 children on the second day, 3 children on the third day and 1 child on the fifth day. Unfortunately, the limited number of cases does not allow any statistical comparison.

### Adverse events and clinical tolerability

Only 2 patients reported mild treatment-related adverse events: diarrhoea and vomiting (1 child in the PEG-only group) with unlikely relationship, for which the treatment was interrupted for two days, and abdominal pain (1 child in the PEG-EL group) with probable relationship, for which the treatment was stopped.

The PEG-only group was better in terms of tolerability. The comparison is significant for the symptom of nausea (p=0.003), but not for abdominal discomfort (Table 3).

**Table 3:** Tolerability

	**PEG-only**		**PEG-EL**	
Number of evaluable patients	49		42	
Nausea				
No episodes	48	(98%)	33	(79%)
1 or 2 episodes	1	(2%)	9	(21%)
		*p*=0.003		
Abdominal discomfort				
No episodes	40	(82%)	27	(64%)
1 or 2 episodes	8	(16%)	10	(22%)
3 to 5 episodes	1	(2%)	3	(8%)
more than 5 episodes	0	(0%)	2	(6%)
		*p*=0.155		

### Acceptability and compliance

Overall, 96% of patients in the PEG-only group *vs*. 52% of patients in the PEG-EL group showed no difficulties associated with the treatment (p < 0.001).

Also the taste of the PEG-only product was evaluated as better than PEG-EL (p < 0.001) (Table 4).

**Table 4:** Patient acceptability

	**PEG- only**		**PEG- EL**	
Number of evaluable patients	49		42	
Difficulty in administration				
No difficulty	47	(96%)	22	(52%)
Mild difficulty	1	(2%)	17	(40%)
Severe difficulty	1	(2%)	3	(37%)
		*p*<0.001		
Taste/palatability				
good / very good	21	(43%)	1	(2%)
not good – not bad	27	(55%)	30	(71%)
Bad / very bad	1	(2%)	11	(26%)
		*p*<0.001		

The difference between the percentage of subjects who took >80% of the prescribed dose (threshold established to determine the compliance of subjects) is in favour of the PEG-only group (98% *vs*. 88%) but does not reach the usual significance level (p = 0.062).

## Discussion

We carried out a randomized head-to-head comparison between a PEG-only laxative and a PEG-EL formulation for resolution of faecaloma and treatment of constipation in children referred to paediatric departments.

According to common criteria, both agents were effective, and well tolerated in achieving satisfactory relief of constipation and resolution of faecal impaction in children. However, our study aimed at evaluating whether there are differences that are worth taking into consideration when selecting an appropriate laxative therapy in clinical practice. The choice of a laxative also depends on other parameters such as palatability, ease of administration and compliance which may even have an impact on the treatment outcome [21]. Our study is important because it provides a detailed comparison of two PEG-laxatives in the treatment of chronic constipation in children.

In this study children in the PEG-only group had higher stool frequency and a greater number of days with stool than those in PEG-EL group. Stool frequency alone is not necessarily a measure of the clinical efficacy and other variables are sometimes considered (stool consistency, difficult or painful defecation) especially for adults.

The small sample of children with faecal impaction did not allow a proper statistical comparison; PEG-only appeared to be slightly faster than PEG-EL in disimpaction

Based on their efficacy and safety profile, PEG-based laxatives have become the most popular agents for the treatment of constipation in adults [5] as well as in children [22-24].

PEG acts as an osmotic agent which increases water content of stools and hence stimulates colonic peristalsis and transit of softened stools making bowel evacuation easier [25]. A great advantage, compared with other osmotic agents such as lactulose, is that PEG is not absorbed and metabolized by human intestinal enzymes and colonic bacteria.

In general, PEG formulations can be associated, to a lesser extent, with nausea, bloating and bad taste which can cause problems with compliance. In our study, the PEG-only product was better tolerated as its use was almost free from nausea, which occurred in about 1 out of 5 children treated with PEG-EL, while we were unable to detect differences for bloating, which was only occasionally reported on the patient diary and physician questioning.

We evaluated the use of PEG products in children aged 2 years and older but other investigators have shown that PEG-only formulation is also well tolerated in infants and toddlers [26-28].

In this study, the PEG-only formulation has been shown to have better palatability and ease of administration than the PEG-EL formulation. This may be due to the fact that the PEG-only agent is free of saline or flavouring agents.

This is a very important aspect, particularly in paediatric age, because a higher adhesion to treatment may contribute to a more effective outcome especially in clinical practice where carers are generally less motivated than those in clinical trials. A trend toward a better compliance was actually found in the PEG-only group.

This study confirms that PEG-based agents are also effective for faecal disimpaction. This has an important clinical implication as it confirms that, in most situations, oral PEG may replace rectal laxatives (suppositories and enemas) which have been used to evacuate stools from rectum and distal sigmoid colon but are inconvenient for children and their careers. It is worth adding that in our experience, resolution of faecal impaction with oral PEG was achieved in a relatively short time with complete relief of agitation which is a frequent feature in clinical practice.

As the two treatments are different in appearance, dosage instruction and taste, our study could not be carried out as double blind. We are aware that this is a potential source of bias, especially for subjective evaluations. However, the PEG-only formulation was constantly superior for most objective and subjective parameters, including the frequency of stools which is considered the most objective efficacy measure in constipation.

PEG is the major active ingredient in PEG-EL as the electrolytes (i.e. sodium chloride, potassium chloride and sodium bicarbonate) do not exert any pharmacological activity. PEG-EL when dissolved in the specified amount of water is iso-osmotic relative to plasma which therefore reduces the potential loss of water accompanying electrolytes from plasma. As a matter of fact, both PEG formulations with and without electrolytes have long post-marketing safety experience with no systemic adverse effects or effects on electrolyte balance. The probable explanation for the better efficacy of the PEG-only formulation is that it contains a higher amount of PEG per single dose as compared to PEG plus electrolytes. At the doses recommended by the respective prescribing information which have been used in our study, the efficacy of two PEG-formulations for the treatment of chronic constipation in children can not be considered equivalent. It is worth noting that the dose of PEG-only formulation is determined according to child weight while PEG plus electrolytes according to age class. The PEG-only formulation allows for a more accurate determination of dose and in addition it can be taken with everyday beverages. In order to avoid confusion, in our study the single dose of PEG-only was dissolved in 125 ml of water or other fluids

Prolonged use of these laxatives is not recommended, but in some circumstances there is a need to use laxatives for a long term. Some studies have indicated that PEG-EL and PEG-only do not induce tolerance and there is no need to increase the dose during long term use [15].

## Conclusion

This randomized comparative study suggests that the PEG-only laxative is effective and well tolerated for faecal impaction and chronic constipation in children and that it may be superior to the PEG-EL formulation in terms of tolerability and ease of administration.

Further studies in children are needed to evaluate the efficacy, tolerability and compliance of PEG-only formulation in longer term studies.

## Abbreviations

PEG: Polyethylene glycol; PEG-EL: Polyethylene glycol – Electrolytes.

## Competing interests

The experimental products were supplied by PROMEFARM srl, Milano (Italy).The study was partially funded through a grant from Promefarm to our Institutions. The funding sources had no role in the study design, data collection, data interpretation, data analysis, or writing the manuscript.

## Authors’ contributions

FS and SC developed the study design and drafted the manuscript. SV, EM, GDN and SO were involved in recruitment of patients and conduct of study. Analysis of data was made by Antonio Rinaldi PhD, statistician consultant. All the authors read and approved the final manuscript.

## Pre-publication history

The pre-publication history for this paper can be accessed here:

http://www.biomedcentral.com/1471-2431/12/178/prepub
